# Pulmonary Atheroma

**Published:** 1909-10-23

**Authors:** 


### Medicine.

### PULMONARY ATHEROMA.

Atheroma of the pulmonary arteries and arterioles is a condition that attracts comparatively little notice, and yet it is one which has certain clinical bearings of importance. In the first place, it has practically no relation whatever to atheroma of the aorta or of systemic arteries. Ordinary systemic atheroma is a disease of the second half of life, often associated with high arterial blood-pressure, fibrous degeneration of the kidneys, and arterio-sclerosis. In patients affected by it the pulmonary arterioles are often perfectly free from any atheromatous changes at all. In the second place, there is one cause of pulmonary atheroma that far outweighs all the others put together, and that is mitral stenosis. In long-standing cases of mitral stenosis there is nearly always great thickening and atheroma of all the branches of the pulmonary artery, due no doubt to the pressure within those branches being kept continually at a much higher level than normal, owing to the right ventricle continuing to pump blood forward with even more than its usual force, whilst the narrowing of the mitral valve prevents the blood thus pumped into the pulmonary circulation from escaping forward into the left ventricle so quickly as it should. The result of the continual increased strain upon the walls of the pulmonary arterioles is to thicken them, particularly their inner coats. Thirdly, if we remember that there are two main factors in the production of intra-vascular thrombosis—namely, slowing of the blood-stream upon the one hand, and alteration of the lining wall of the vessel upon the other—it will be clear that we have both these essentials in the pulmonary arterioles in cases of mitral stenosis; hence it is not surprising that hæmoptysis is a common symptom of the latter disease, due, in part at least, to the production of thrombotic infarcts. An infarct is, of course, the name of the wedge-shaped mass that one is familiar with in the post-mortem room, but it is too often assumed that this infarct is due in all cases to an *embolus*. This is by no means the case. An infarct, when the blood supply of an organ allows of its being produced at all, results simply from the occlusion of an end-artery; it matters not how that occlusion takes place—it may be by *thrombosis*, or it may be by *embolism*. There are, therefore, thrombotic infarcts on the one hand, and embolic infarcts upon the other; both look identical in the post-mortem room, but in cases of mitral stenosis it is far commoner for pulmonary infarcts to be thrombotic, not embolic, and the reason for this is the extensive atheroma of the pulmonary arterioles that mitral stenosis gives rise to.

A domestic servant, aged twenty, came into hospital on April 22, giving a history that she had had none of the infectious diseases of childhood and no rheumatic fever, but that for a long time past she had been very short of breath if she ran or played with children, or even walked rapidly. Three years before admission an attack of dyspnœa had been followed by hæmoptysis, cyanosis of the extremities, and slight œdema of the ankles, as the result of which she had been taken to a hospital, where mitral stenosis was diagnosed. She remained as an in-patient for four weeks, and then returned to her duties very greatly better. From that time onwards, however, she had been subject to repeated cardiac attacks, cough, and hæmoptysis. Five months before her final admission there had been a particularly severe hæmoptysis, during which three large tumblersful of blood were coughed up. This relieved her greatly for the time being, but a month later hemiplegia developed, together with complete inability to keep any food in the stomach on account of persistent vomiting. The physical signs indicated mitral stenosis, and within a short time the poor girl was dead.

At the autopsy the chief points noted were extreme mitral stenosis and changes secondary to that. The lungs looked natural externally; but on cutting up the pulmonary artery it was found to be lined by atheromatous plaques; and when it was followed along as far as it could be into the lungs, no branch of it could be found that was not extensively affected by atheroma. There was no similar atheroma of the aorta, and there is no need to note all the changes that the mitral stenosis had produced in the other viscera.

Under the microscope the tunica intima of the main arteries was found to be greatly thickened, particularly at the site of the patches, which to the naked eye formed yellow projections into the lumen. Beneath the thickened patch the tunica media seemed but slightly altered, the elastic fibres were well marked, even more so than normal, and here and there, particularly close to the tunica intima, the muscle fibres may have stained less well than they should do. In branches of medium size the changes were even plainer and more widespread. In sections stained with orcein, it could be clearly seen that the local thickenings of the tunica media consisted of two parts—an inner, which was amorphous, and an outer, which abutted on the tunica media, and consisted of a fine reticulum of very short elastic fibrils enclosing the ill-defined nuclei of degenerating muscle fibres. In many places the tunica media immediately under this showed extensive changes also.

In the smallest arterioles the twisting membrane of elastic tissue was nearly always at least doubled, and often it was represented by several layers, in marked contrast to the normal.

---

There can hardly be a doubt that purely mechanical causes, the chief of which is increased intra-arterial pressure, lie at the root of the atheroma of the pulmonary artery in cases of mitral stenosis. Clinical evidence of this increased pressure is afforded by the great accentuation, with or without reduplication of the pulmonary second sound. It would seem likely that systemic atheroma is also due quite as much to mechanical as to toxic causes.

